# The New Era of Physio-Logging and Their Grand Challenges

**DOI:** 10.3389/fphys.2021.669158

**Published:** 2021-03-30

**Authors:** Andreas Fahlman, Kagari Aoki, Gemma Bale, Jeroen Brijs, Ki H. Chon, Colin K. Drummond, Martin Føre, Xavier Manteca, Birgitte I. McDonald, J. Chris McKnight, Kentaro Q. Sakamoto, Ippei Suzuki, M. Jordana Rivero, Yan Ropert-Coudert, Danuta M. Wisniewska

**Affiliations:** ^1^Fundación Oceanográfic de la Comunitat Valenciana, Valencia, Spain; ^2^Department of Marine Bioscience, Atmosphere and Ocean Research Institute, The University of Tokyo, Kashiwa, Japan; ^3^Department of Physics and Department of Engineering, University of Cambridge, Cambridge, United Kingdom; ^4^Hawai'i Institute of Marine Biology, University of Hawai'i at Manoa, Manoa, HI, United States; ^5^Biomedical Engineering, University of Connecticut, Storrs, CT, United States; ^6^Biomedical Engineering, Case Western Reserve University, Cleveland, OH, United States; ^7^Department of Engineering Cybernetics, Norwegian University of Science and Technology, Trondheim, Norway; ^8^Department of Animal and Food Science, Autonomous University of Barcelona, Barcelona, Spain; ^9^Moss Landing Marine Labs at San Jose State University, Moss Landing, CA, United States; ^10^Sea Mammal Research Unit, University of St. Andrews, Scotland, United Kingdom; ^11^Akkeshi Marine Station, Field Science Center for Northern Biosphere, Hokkaido University, Akkeshi, Japan; ^12^Rothamsted Research North Wyke, Okehampton, United Kingdom; ^13^Centre D'Etudes Biologiques de Chizé, La Rochelle Université, UMR7372, CNRS, France; ^14^Department of Biology, University of Southern Denmark, Odense, Denmark

**Keywords:** bio-logging, bio-telemetry, physiology, heart rate, accelerometers, welfare, conservation

## Introduction

The field of bio-sensing refers to studies where the physiology of an animal, its behavior and movement, as well as the characteristics of the environment it moves in, is measured either by electronic sensor-carrying devices that store the data (bio-logging), or those that transmit the data directly (bio-telemetry).^1^ One of the first bio-sensing studies was conducted over 80 years ago with the attachment of a capillary tube to a fin whale (*Balaenoptera physalus)* to assess the dive depth of a free-ranging marine mammal (Scholander, 1940). In humans, the stethoscope was developed by Rene Laennec in 1819 as the first non-invasive heart monitor, which solved the challenge of listening to the heart by placing an ear on the patient's chest (not always welcome in the Victorian era) (Roguin, 2006). Quickly the system found new uses eventually leading to a shift from subjective to objective data about the internal body. The field of bio-sensing has since increased exponentially and revolutionized our understanding of animal ecology. With the technological development of miniaturized sensors, numerous studies of movement ecology, behavior, and communication in a diverse range of animals (e.g., species of fish, reptiles, birds and mammals) have been reviewed in (Frost et al., 1997; Davis, 2008; Ropert-Coudert et al., 2009a; Rutz and Hays, 2009; Swain et al., 2011; Hussey et al., 2015; Wilmers et al., 2015; Endo and Wu, 2019; Börger et al., 2020; Wassmer et al., 2020). While determining the physiological limits and plasticity of a species is essential for understanding its ecology and evolution, studies that measure the physiological responses of free-ranging animals (i.e., physio-logging) have not seen the same exponential increase, even though physiological questions were at the origin of the use of data loggers in seminal work done by field physiologists such as Gerry Kooyman, Paul Ponganis, Warren Zapol, and Patrick Butler (Butler and Woakes, 1979; Falke et al., 1985; Kooyman, 1985; Ponganis et al., 1991). The slower growth of the physio-logging field could be due to the commercial unavailability of physiological sensors, or that the available sensors were too large, based on static-technologies, or required specialized surgical training and extensive knowledge of the anatomy and physiology of the animal for successful implantation. Despite these challenges, studies using bio-sensing tools have renewed the interest in physio-logging and attempted to understand the physiology of an animal through inference from their behavior (Wilson et al., 2002; Hooker et al., 2009; Goldbogen et al., 2011, 2019b; Kolarevic et al., 2016; Føre et al., 2018b; Quick et al., 2020).

Physio-loggers have recently been used on farmed animals (livestock) to record physiological variables (e.g., body temperature, respiration and heart rates) in order to monitor water intake, the occurrence of diseases, energy expenditure in grazing activities, and effect of diet on body temperature under cold and warm conditions (Brosh et al., 2006; Eigenberg et al., 2008; AlZahal et al., 2011; Arias et al., 2011; Aharoni et al., 2013; Cantor et al., 2018). In the human arena – where early bio-telemetry approaches were born – technological advances such as movement sensors initially allowed anyone with a “smartphone” or “smartwatch” to assess their daily energy consumption, leading to the so-called “quantified health” movement (Scully et al., 2012). Indeed, subsequent development of non-invasive sensing (photoplethysmography) enabled new and exciting possibilities to track health and fitness in a large number of people (Dörr et al., 2019; Seshadri et al., 2020). In addition, recent developments in wearable medical and nanotechnology, with increased battery life, storage capacity and a range of sensors have increased our ability to study physiological function both non-invasively and continuously over months and years (Kang et al., 2016; Kaidarova et al., 2018, 2019; Lee et al., 2019; Lazaro et al., 2020). Thus, tools capable of measuring a range of important and informative physiological parameters are now available, and are continuously being improved and adapted to work on an increasing range of species. These developments will revolutionize the capacity to measure and assess the physiology of animals and humans over extended periods of time, which will allow a comprehensive evaluation of the physiological function of animals in their natural environment. This new era of physio-logging will enable long-term studies to better understand fundamental physiological function, health, welfare or well-being of animals and humans, as well as their responses to environmental and/or anthropogenic changes.

## The Paradigm Shift Challenge

Much of what we know about animal physiology has been obtained by measuring physiological parameters on captive, semi-captive, or restrained animals including measures of heart rate, blood flow, blood chemistry, blood gases, and metabolic rate (Berkson, 1967; Kooyman et al., 1970; Kooyman and Campbell, 1972; Kooyman and Sinnett, 1982; Lutcavage et al., 1989; Ponganis et al., 1990; Reed et al., 1994, 2000; Gräns et al., 2010; Kang et al., 2016; Brijs et al., 2018; Berenbrink, 2021; Svendsen et al., 2021). Unfortunately, in such situations, it is difficult to assess the magnitude of potential confounding factors such as stress or manipulation on the measured physiological variable. In recent years, there has been a focus on measuring physiology in free-ranging animals. For example, trained animals that are desensitized to the experimental procedures have been used to study diving energetics, cardiorespiratory and vascular physiology, and cerebrovascular physiology (Elsner, 1965; Olsen et al., 1969; Ridgway and Howard, 1979; Williams et al., 1993; Hurley and Costa, 2001; Fahlman et al., 2008, 2019, 2020a,b; Mortola and Sequin, 2009; Rosen and Trites, 2013; Worthy et al., 2013; Elmegaard et al., 2016, 2019; Takei et al., 2016; McKnight et al., 2019; Meir et al., 2019; Pedersen et al., 2020; Blawas et al., 2021). As bio-logging technologies have advanced, physiological parameters such as heart rate, respiration rate, and blood O_2_ have even been measured in free-ranging fish, reptiles, birds, and mammals (Falke et al., 1985; Ponganis et al., 1991; Thompson and Fedak, 1993; Southwood et al., 1999; Andrews et al., 2000; Froget et al., 2004; Ropert-Coudert et al., 2006, 2009b; Meir et al., 2009; Yamamoto et al., 2009; Meir and Ponganis, 2010; McDonald and Ponganis, 2013, 2014; Sakamoto et al., 2013; Duriez et al., 2014; Goldbogen et al., 2019a; McKnight et al., 2019, 2021a,b; Sumich, 2021). There is a parallel shift in the paradigm of physiological monitoring of humans. Whereas once health monitoring was exclusively a physician-based in-patient activity, the emergence of health and fitness wearables has led to the so-called “quantified self” movement, where patients are producing data in support of diagnostics (Patel and Tarakji, 2021).

## Technological Development

Progress on medical sensing technology has increased significantly. A wide range of physiological monitoring technologies are now available and are setting the stage from which physiological bio-sensing could profit immensely. For instance, virtually anyone with a “smartphone” or “smartwatch” can now assess their daily calorie expenditure as sensors within the phone can estimate the number of steps taken or distance moved. Similarly, researchers have applied this principle to free-ranging animals and are able to derive an estimate of energy expended in the wild via a measure of dynamic body movements measured by animal-embarked accelerometers (Wilson et al., 2006, 2020; Gleiss et al., 2011) that correlates well with other direct measures of energy expended even in wild animals (Elliott et al., 2013; Jeanniard-Du-Dot et al., 2017; Hicks et al., 2020). Phonospirometry (i.e., the use of the breath sound to estimate respiratory flow) is being used to perform lung function testing in both humans and animals (Sumich and May, 2009; Larson et al., 2012; Sumich, 2021; Van Der Hoop et al., 2021). In addition, the ongoing development of wearable medical sensors that can detect glucose levels, estimate heart rate via waterproof ECG electrodes (Reyes et al., 2014; Noh et al., 2016) or assess blood flow/volume changes and/or blood oxygen saturation changes provide a particularly exciting avenue for future research (Bockstaele et al., 2014; McKnight et al., 2019, 2021a,b). These technological advancements open up enormous possibilities as they will enable investigating the physiological function in freely moving, and even free-ranging animals, with minimal disturbances. Further, the development of fully bioresorbable microchip technologies capable of measuring a variety of physiological parameters (Kang et al., 2016) could offer opportunities to measure new, fine-scale physiological metrics in free-moving and free-ranging animals. Thus, long-term data sets on movement, married with physiological data could become available, contributing essential components to frameworks that assess the consequences of environmental and/or anthropogenic impacts such as Population Consequences of Disturbance (PCoD, Booth et al., 2014; Pirotta et al., 2018), as well as to develop a fundamental understanding of the physiology of a diverse range of species.

## Analytical Development

The collection of long-term and/or high-resolution data sets is likely to result in analytical challenges. For example, ECG collection sampled at 200 Hz over a whole year results in 6.3 billion data points. While ECG could be reduced to instantaneous heart rate (i*f*_H_) (Sakamoto et al., 2021), normal statistical tools, such as comparison of means or medians are not applicable and are likely to result in erroneous conclusions. More sophisticated analytical methods, including signal processing or time-series analysis, will have to be developed and introduced to deal with a growing number of studies that focus on physiological function and eco-physiology. There has recently been a rapid growth in analytical techniques in bioinformatics, where new tools and databases have been developed to handle the large data sets that result from sequencing the genome of various species and to evaluate gene networks and differential changes in molecular products. A similar exponential growth has been seen within data processing methods based on Artificial Intelligence (AI) and Machine Learning (ML). These methods are used in several different fields today, especially when data sets are too large and/or complex to handle through conventional means, and it is likely that they prove useful for processing datasets from bio-sensors. Although many AI/ML methods are “a black box,” in the sense that they do not describe the mechanistic links between input and output (e.g., environmental and/or anthropogenic changes and sensor output in this case), they could be useful for compressing and condensing large data sets and identifying unknown relationships between inputs and measured features (Rasheed et al., 2020).

## Conclusion

In the last 40 years, the field of bio-sensing has provided important information about the ecology and behavior of wild animals, largely focusing on describing *where* they go and *what* they do there. Animal tracking studies have substantially improved the knowledge of movement patterns and drivers of movement in marine, terrestrial and avian species. However, the rapid development and miniaturization of bio-sensing electronics capable of measuring a raft of physiological variables present innovative and exciting tools that will revolutionize this field of research and usher in a new era of physio-logging. These technologies will allow us to comprehensively evaluate *how* and *why* animals make the journeys they do (e.g., bar-headed geese flying over the Himalayas, Cuvier's beaked whales diving to 3000 m for over 3 h; Hawkes et al., 2013; Quick et al., 2020). Such studies will provide a foundation for understanding how animals may respond to alterations in the environment and the physiological boundaries for survival. Physio-logging can also provide the necessary tools for conservation management, which will contribute toward reducing the impacts of anthropogenic disturbances on species, communities and even ecosystems. For example, assessment of stress levels, such as measuring corticosterone or heart rate may help evaluate the impact of anthropogenetic disturbance (Miksis et al., 2001). Furthermore, physio-logging is also likely to provide an important diagnostic tool for evaluating the well-being and welfare of farmed terrestrial and aquatic animals. These technologies can provide a unique opportunity for health monitoring via an “animal-eye” view of the conditions that farmed animals experience in human care on a day-to-day basis, enabling a better understanding of how to address the challenges faced by industries attempting to produce a profitable, ethical and environmentally sustainable product (Berckmans, 2004; Føre et al., 2018a; Brijs et al., 2021). Many of the responses evaluated in these managed settings could also be translated to wild animals given the clear link between physiology and welfare (Gregory, 2004; Baird et al., 2016; Føre et al., 2018a; Svendsen et al., 2021). Finally, physio-logging is likely to promote improved health and wellness in humans, where early detection of disease allows improved treatment outcome.

As we enter a new age in the study of physiology of animals and humans living in complex environments, the Physio-logging journal aims to provide a forum where scientists, and conservation practitioners among others, can share knowledge on how modern sensing technology and analytical approaches can be used to understand physiological function and health of animals and humans.

## Author Contributions

All authors listed have made a substantial, direct and intellectual contribution to the work, and approved it for publication.

## Conflict of Interest

AF was employed without salary by the company Global Diving Research Inc. The remaining authors declare that the research was conducted in the absence of any commercial or financial relationships that could be construed as a potential conflict of interest.

## Footnotes

^1^Recent advances combine the logging and transmission of data using AI and machine learning approaches to process data on-board the logger and transmit either subsets of data or information derived from the primary data recorded.
